# Modified Singular Spectrum Decomposition and Its Application to Composite Fault Diagnosis of Gearboxes

**DOI:** 10.3390/s19010062

**Published:** 2018-12-24

**Authors:** Junyuan Wang, Xiaofeng Han, Zhijian Wang, Wenhua Du, Jie Zhou, Jiping Zhang, Huihui He, Xiaoming Guo

**Affiliations:** College of Mechanical Engineering, North University of China, Taiyuan 030051, China; wjy@nuc.edu.cn (J.W.); feng5524309@163.com (X.H.); dwh@nuc.edu.cn (W.D.); zj_zhongb@163.com (J.Z.); zhjp-0000@163.com (J.Z.); hehuihui124@163.com (H.H.); guoxiaoming1113@163.com (X.G.)

**Keywords:** Gearbox composite fault, algorithm, fault diagnosis, modified singular spectrum decomposition

## Abstract

Under the strong noise environment, the composite fault signal of gearbox is weak, which makes it difficult to extract fault features. For this problem, based on noise-assisted method, we propose a novel method called Modified Singular Spectrum Decomposition (MSSD). Singular Spectrum Decomposition (SSD) has many advantages such as high decomposition precision and strong ability to restrain mode mixing, etc. However, the ability of SSD to extract a weak signal is not ideal, the decomposition results usually contain a lot of redundant noise and mode mixing caused by intermittency, which is also a troubling problem. In order to improve the decomposition efficiency and make up for the defects of SSD, the new method MSSD adds an adaptive and particular noise in every SSD decomposition stage for each trial, and in addition, whenever the input signal is decomposed to obtain an intrinsic module function (IMF), a unique residual is obtained. After multiple decomposition, the average value of the residual is used as input to the next stage, until the residual cannot continue to decompose, which means that the residual component has, at most, one extreme value. Finally, analyzing simulated signals to explain the advantages of MSSD compared to ensemble empirical mode decomposition (EEMD) and complete ensemble local mean decomposition with adaptive noise (CEEMDAN). In order to further prove the effectiveness of MSSD, this new method, MSSD, is applied to the fault diagnosis of an engineering gearbox test stand in an actual engineer project case. The final results show that MSSD can extract more fault feature information, and mode mixing has been improved and suffers less interference compared to SSD.

## 1. Introduction

In recent years, gearbox composite fault diagnosis has attracted wide attention [1,2]. When the internal parts of the gearbox fail, it will cause the performance of the complete mechanical equipment to decline, and further affects the working status of the whole production system [3]. Thus, it is of great significance to take effective measures for fault diagnosis at gearbox [4,5,6,7]. Due to the high relativity between vibration signal and operating status of gearbox, the vibration analysis based on this method of processing signal is used in fault diagnosis widely and is important [8,9,10].

In recent years, there are many methods of fault extraction, such as spectrum analysis, artificial intelligence-based diagnosis [11,12,13,14,15,16], model-based identification [17], wavelet analysis [18,19], and higher order spectra analysis [20,21,22,23]. In 1998, N. E. Huang [24] proposed the empirical mode decomposition method (EMD), it can decompose any composite signal into a set of intrinsic module function (IMF). However, it will occur in the mode mixing when the distribution of extreme points in the signal is uneven, which will affect the results of EMD decomposition. Wu and Huang proposed the ensemble empirical mode decomposition (EEMD) in 2009. EEMD is based on EMD method, and EEMD uses the uniform distribution of Gaussian white noise to mix white noise into the input signal, and makes the original signal has continuous at different time scales, so that mode mixing phenomenon can be alleviated [25]. EEMD can decompose composite signals into a series of IMF, make different frequency distribute into different IMFs, so it can improve the results of the decomposition and reduce noise. While EEMD alleviates the mode-mixing problem to some extent, there are still two difficulties to overcome. The first is that the results of EEMD decomposition contains redundant noise components, which require a lot of experiments to eliminate redundant noise components, but this process is time consuming. The second, adding the Gaussian white noise into the original signal, because the randomness of Gaussian white noise, different experiments may produce a different number of signal components, which makes it difficult to take the ensemble mean in the final result. In 2010, N. E. Huang proposed the Complementary ensemble empirical mode decomposition (CEEMD) [26], the principle of this method is to add pairs of complementary white noise to the input signal, which effectively eliminates residual noise due to the characteristics of white noise. Compared to EEMD, CEEMD uses fewer ensemble trials, but the problem is that it is difficult to take the ensemble mean, which remains unsolved. In the recent years, Torres [27] proposed the complete ensemble local mean decomposition with adaptive noise (CEEMDAN), which is based on the EEMD method and solves the problem of EEMD. In 2014, Colominas [28] continued to modify CEEMDAN method, and it solves some of the problems that redundant IMFs may occur in the earlier stage of decomposition. Due to the advantage of CEEMDAN, in 2017, Lei and others [29,30] used it to diagnose various mechanical faults and achieved satisfactory results, but CEEMDAN still has some problems that need to be solved. For example, the decomposition results still exist in mode-mixing and increase the number of iterations of the algorithm in order to reduce mode-mixing, and the computation time will be too long.

In 2014, P. Bonizzi [31] proposed a novel fault signal decomposition method, called singular spectrum decomposition (SSD). This is a new iteration time series decomposition method and it is created on the basis of singular spectrum analysis (SSA) [32,33,34]. SSD has already been successfully applied in signal processing, for example, P. Bonizzi makes SSD applicable in tide and seaquake data processing analysis and has achieved satisfactory results. In the SSD method, its principle is to reconstruct a single component signal from high frequency to low frequency adaptively, SSD provides a new method for fault diagnosis and signal processing. Similar to EMD, SSD decomposition is based on extracting signal components associated with various intrinsic time scales. Compared to EMD, mode mixing of SSD has been alleviated in a way, and SSD can separate the intermittent components at the transition point accurately. However, there are still some problems in SSD decomposition, such as redundant components and mode mixing is generated. Under strong noise, the fault information is easily submerged and fault feature frequency is difficult to extract.

Inspired by the previous studies, we propose a new method to solve the existing problems of SSD. An IMF of adaptive amplitude Gaussian white noise pre-processed by the SSD is added at each decomposition stage for each trial. In addition, when an IMF is separated, we also get a unique residual, the pre-processed white noise plus the obtained residual is then used to form a new signal as a new input signal for the next stage. Therefore, this new method has modes with better spectral separation and the decomposition results have less residual noise, the number of sifting iterations is also less than other methods.

The article is arranged as follows. In the Section 2, we introduce the basic principles of SSD and MSSD briefly. The Section 3 provide the comparative and research of the EEMD, SSD, and MSSD methods through decomposing simple simulate signal. Section 4 compares CEEMDAN, SSD, and MSSD through the decomposition result of composite simulate signal, and analyzes the result. In Section 5, the MSSD method is used to deal with the fault of gearbox in an actual engineer project case, and analyzes the result. Finally, Section 6 summarizes the whole research and gives a prospect for the future.

## 2. Principles of the Algorithm 

### 2.1. The Principle of SSD

The principle of the SSD algorithm is to extract the signal components one by one by an iterative method, and to decompose the original signal into meaningful signal components. The specific algorithm of SSD is as follows:

Step 1: Building a time series x(n), and the length of this time series is *N.* Then, given an embedding dimension *M*, a (*M* × *N*) matrix *X* is created, we can get the *i*-th row as: $X_{i}=(x(i),\cdots x(N),x(1),\cdots x(i-1))$, where i=1,⋯,M, and therefore, $X={\left[x_{1}^{T},x_{2}^{T},\ldots ,x_{M}^{T}\right]}^{T}$. For example, building a time series x(n)={1,2,3,4,5}, and selecting an embedding dimension M = 3, the corresponding trajectory matrix is as follows:

(1) $$X=\left[\begin{array}{cc} \begin{array}{ccc} 1 & 2 & 3\\ 2 & 3 & 4\\ 3 & 4 & 5 \end{array}| & \begin{array}{c} \begin{array}{cc} 4 & 5 \end{array}\\ \begin{array}{cc} 5 & 1 \end{array}\\ \begin{array}{cc} 1 & 2 \end{array} \end{array} \end{array}\right]$$

The left side of the matrix corresponds to the matrix *X* used in the standard SSA algorithm. In order to enhance the oscillation component in the original signal and to make the energy of the residual component after iteration show a decreasing law, the three elements in the lower right corner of the matrix must be appended to the upper right-hand corner of the left block. The new matrix constructed is as follows:

(2) $$X=\left[\begin{array}{cc} \begin{array}{ccc} & & 1\\ & 1 & 2\\ 1 & 2 & 3\\ 2 & 3 & 4\\ 3 & 4 & 5 \end{array}| & \begin{array}{cc} 4 & 5\\ 5 & *\\ {}* & * \end{array} \end{array}\right]$$

Step 2: Selecting the embedding dimension in the *j*-th iteration. The power spectral density (PSD) is calculated, where PSD is derived from the residual components $v_{j}$ in the *j*-th iteration:

(3) $$v_{j}(n)=x(n)-\sum_{k=1}^{j-1}v_{k}(n)\;(v_{0}(n)=x(n))$$

Estimating the frequency $f_{\max}$ in its PSD related to the dominant peak. If the normalized frequency $f_{\max}/F_{s}$ (with $F_{s}$ being the sampling frequency) is in the first iteration, it is less than a given threshold (set to 0.01 in the experiment), then given M is N/3. Otherwise, and for iterations *j* > 1, the embedding dimension is: $M=1.2\frac{F_{S}}{F_{MAX}}$.

Step 3: In the first iteration, reconstructed the *j*-th component series, if a considerable trend is found, using only the first left eigenvectors and the right eigenvectors to obtain $g^{\left(1\right)}(n)$, in this case, $X_{1}=\sigma_{1}u_{1}v_{1}{}^{T}$, and $g^{\left(1\right)}(n)$ is obtained from diagonal averaging of $X_{1}$. Otherwise, when the iterations *j* > 1, a subset $I_{j}\left(I_{j}=\left\{i_{1},\ldots ,i_{p}\right\}\right)$ is created by selecting a feature vector group whose left eigenvector $\left[f_{\max}-\delta f,f_{\max}+\delta f\right]$ has the largest dominant frequency in the spectral range. Then, through the diagonal averaging of the matrix $X_{Ij}=X_{i1}+\ldots +X_{ip}$, it is possible to reconstruct the corresponding component sequence. Where δf represents half of the main peak width in the PSD sequence and needs to be estimated from the PSD of $v_{j}\left(n\right)$. 

Step 4: Establish stopping criterion, calculate a new residual when ${\tilde{g}}^{\left(j\right)}(n)$ is estimated:

(4) $$v^{\left(j+1\right)}(n)=v^{\left(j\right)}(n)-{\tilde{g}}^{\left(j\right)}(n)$$

where $v^{\left(j+1\right)}(n)$ is the input for the next iteration, ${\tilde{g}}^{\left(j\right)}(n)$ is a new component series. Then, calculate the normalized mean squared error (NMSE) between the original signal and the residual:

(5) $$NMSE^{\left(j\right)}=\frac{\sum_{i=1}^{N}{\left(v^{\left(j+1\right)}(i)\right)}^{2}}{\sum_{i=1}^{N}{\left(x(i)\right)}^{2}}$$

When a given threshold (default th = 1%) is more than the NMSE, the decomposition process is stopped. The final result is as follows:

(6) $$x(n)=\sum_{k=1}^{m}{\tilde{g}}^{\left(k\right)}(n)+v^{\left(m+1\right)}(n)$$

In the above formula, m is the number of component series.

### 2.2. Principle of MSSD

Gaussian white noise has the characteristic of uniform distribution in the frequency domain and normal distribution in the time domain. In the experiment, adding Gaussian white noise to the signal can provide a uniformly distributed proportional reference for the signal. The MSSD defines the true modes as the differences between the current input signal and the average of residual components. For each decomposition stage, an IMF of white noise pre-processed by the SSD method with adaptive amplitude is added to the input signal. Then, the input signal with noise is decomposed into an IMF and a residual component. After multiple decomposition, the average of the residual components are taken as the input signal for the next decomposition stage until the stop condition is satisfied (the residual component can not continue to be decomposed, this means that there is at most one extreme value of the residual component). The specific steps of the MSSD algorithm are as follows.

Step 1: Adding white noise to the original signal S(t) to obtain a new signal $s(t)+a_{0}n^{i}(t)$, and operating M-times SSD decomposition on the new signal separately to obtain *M* IMF components of first stage $imf_{1}^{i}(t)$:

(7) $$s(t)+a_{0}n^{i}(t)=imf_{1}^{i}(t)+r_{1}^{i}(t)$$

where $n^{i}(t)$ is the white noise added to the original signal in the *i*-th SSD decomposition, i=1,2,⋯,M, where *M* is the total number of white noise added to the original signal, $a_{0}$ is the amplitude of the white noise, where $a_{0}=\varepsilon_{0}std(s)/std(n^{i}(t))$, $\varepsilon_{0}$ is the standard deviation, and $r_{1}^{i}(t)$ is the first residual component. Average *M* components to get $\bar{imf_{1}(t)}$:

(8) $$\bar{imf_{1}(t)}=\frac{1}{M}\sum_{i=1}^{M}imf_{1}^{i}(t)=s(t)-\frac{1}{M}\sum_{i=1}^{M}r_{1}^{i}(t)$$

It can be seen from Equations (7) and (8) that, due to the influence of white noise, the noise inside $\bar{imf_{1}(t)}$ has been greatly reduced. Then, the first residual component is obtained from Equations (7) and (8):

(9) $$r_{1}(t)=s(t)-\bar{imf_{1}(t)}=\frac{1}{M}\sum_{i=1}^{M}r_{1}^{i}(t)$$

Step 2: The first IMF of white noise by the SSD is added to $r_{1}(t)$ to obtain a new signal $r_{1}(t)+a_{1}E_{1}(n^{i}(t))$, and operating M-times SSD decomposition on the new signal separately to obtain M IMF components of the second stage $imf_{2}^{i}(t)$:

(10) $$r_{1}(t)+a_{1}E_{1}(n^{i}(t))=imf_{2}^{i}(t)+r_{2}^{i}(t)$$

Average M components to get $\bar{imf_{2}(t)}$:

(11) $$\bar{imf_{2}(t)}=\frac{1}{M}\sum_{i=1}^{M}imf_{2}^{i}(t)=r_{1}(t)-\frac{1}{M}\sum_{i=1}^{M}r_{2}^{i}(t)$$

Then, we can get the second residual component:

(12) $$r_{2}(t)=r_{1}(t)-\bar{imf_{2}(t)}=\frac{1}{M}\sum_{i=1}^{M}r_{2}^{i}(t)$$

Step 3: The *k*-th (*k* = 2, 3,⋯) IMF of white noise by the SSD is added to $r_{k}(t)$ to obtain a new signal $r_{k}(t)+a_{k}E_{k}(n^{i}(t))$, and operating M-times SSD decomposition on the new signal separately to obtain M IMF components $imf_{k+1}^{i}(t)$:

(13) $$r_{k}(t)+a_{k}E_{k}(n^{i}(t))=imf_{k+1}^{i}(t)+r_{k+1}^{i}(t)$$

where $a_{k}=\varepsilon_{0}std(r_{k})/std(E_{k}n^{i}(t))$, average M components to get $\bar{imf_{k+1}(t)}$:

(14) $$\bar{imf_{k+1}(t)}=\frac{1}{M}\sum_{i=1}^{M}imf_{k+1}^{i}(t)=r_{k}(t)-\frac{1}{M}\sum_{i=1}^{M}r_{k+1}^{i}(t)$$

Then, we can get the residual component:

(15) $$r_{k+1}(t)=r_{k}(t)-\bar{imf_{k+1}(t)}=\frac{1}{M}\sum_{i=1}^{M}r_{k+1}^{i}(t)$$

It can be seen from Equations (14) and (15) that, due to the influence of white noise, the noise inside $\bar{imf_{k+1}(t)}$ has been greatly reduced.

Step 4: Repeat step 3 until the residual component $r_{k}(t)$ satisfies the stopping condition (the residual component can not continue to be decomposed, that is, the residual component has at most one extreme value). Finally, *k* IMF components are obtained, and the MSSD obtains the residual component R(t), where *k* is the total number of IMFs:

(16) $$R(t)=s(t)-\sum_{k=1}^{K}\bar{imf_{k}(t)}$$

Proof of completeness:

In the decomposition step of MSSD, the left and right sides of Equations (9), (12), (15) and (16) are added, respectively, and the same items on both sides of the equal sign are offset, finally get the following formula:

(17) $$s(t)=\sum_{k=1}^{K}\bar{imf_{k}(t)}+R(t)$$

The above equation can be considered that the original signal s(t) is composed of a series of IMF and residual components, that is to say, the CESSDAN method is complete, and the error of reconstructing the original signal is zero theoretically.

The advantages of MSSD over SSD are as follows. Firstly, the new method adds Gaussian white noise, which pre-processed by SSD in each decomposition period, adding white noise of particular frequency at each decomposition period will help SSD establish a global scale reference and reduce the screening iteration times in each period. Then, SSD decomposes the input signal with noise into one IMF and one residual component. In theory, most of the added white noise and components of the signal with an approximate proportion are extracted into the IMF, so the residual signal components contain very little additional noise. Compared with SSD, IMF extracted by MSSD has a more appropriate proportion and contains less residual noise.

**Figure 1:**
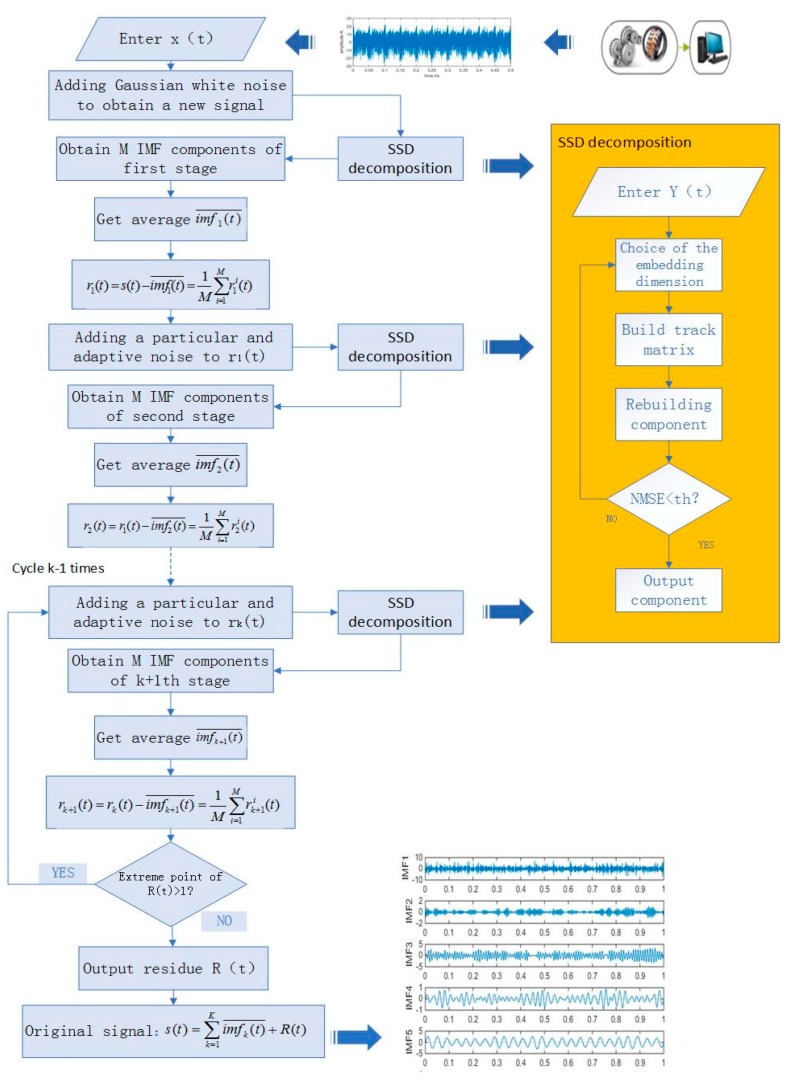
Flow chart of the MSSD.

A flow chart of MSSD is as follows Figure 1:

## 3. Simulation Signal Analysis

### 3.1. Construction of Simulation Signal

**Figure 2:**
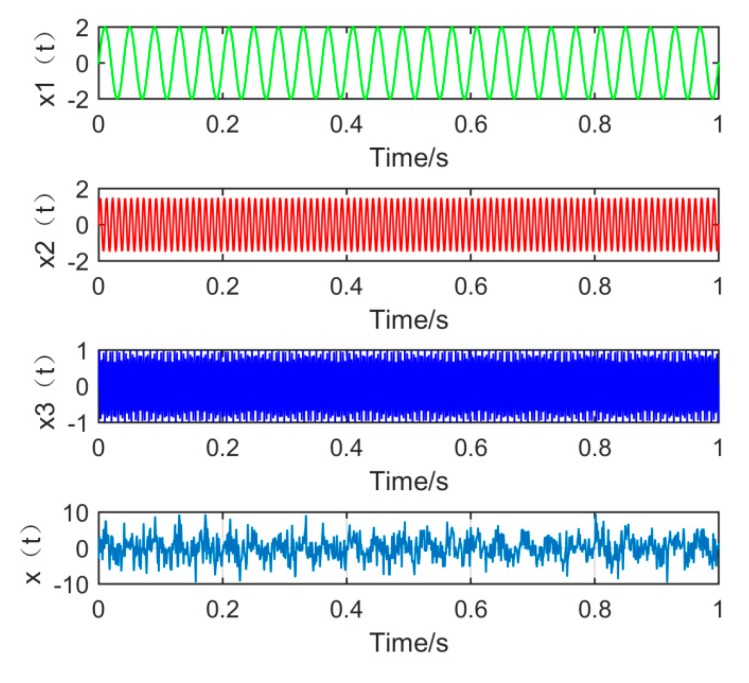
The time domain waveform of each component signal and simulation signal.

First, construct a simulation signal consisting of sinusoidal signals of low, medium, and high frequencies. At the same time, in order to simulate the strong noise environment in engineering practice, noise with amplitude of 1.5 is added to the simulation signal. The simulation signal is as shown in Equation (18):

(18) $$x(t)=x_{1}(t)+x_{2}(t)+x_{3}(t)+1.5\times randn(t),\;\left\{ \begin{array}{l} x_{1}(t)=2\sin(2\pi f_{1}t)\\ x_{2}(t)=1.5\sin(2\pi f_{2}t)\\ x_{3}(t)=\sin(2\pi f_{3}t) \end{array}\right.$$

where $f_{1}=25$ Hz, $f_{2}=100$ Hz, $f_{3}=280$ Hz, set the number of sampling points to *N* = 1000 and the sampling frequency FS = 1000 Hz. The time domain waveform of the signals $x_{1}(t)$, $x_{2}(t)$, $x_{3}(t)$, and the simulated signal *x*(*t*), are shown in Figure 2 below:

### 3.2. Comparison of EEMD, SSD and MSSD

**Figure 3:**
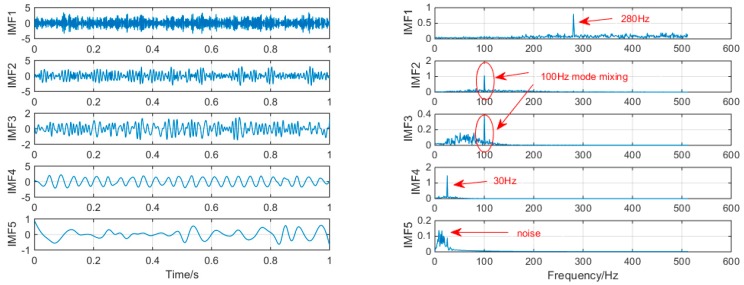
The spectrum of IMFs after EEMD and its corresponding spectrum.

**Figure 4:**
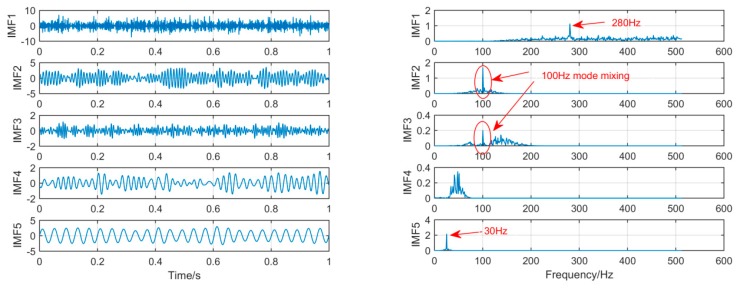
The spectrum of IMFs after SSD and its corresponding spectrum.

**Figure 5:**
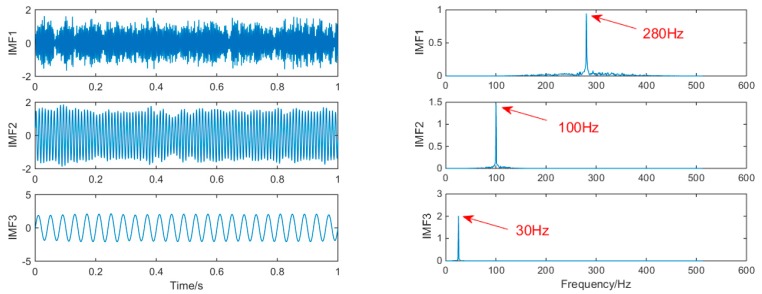
The spectrum of IMFs after MSSD and its corresponding spectrum.

In order to achieve horizontal comparison of the decomposition results of the same simulation signal by different methods. The simulation signal *x*(*t*) will be decomposed by using three signal decomposition methods, EEMD, SSD, and MSSD. In EEMD, the number of cycles N takes 100. The sampling frequency in SSD and MSSD is 1000, and the threshold is chosen to be 0.01. The time domain waveform and spectrum of the decomposition results of the three algorithms are shown in Figure 3, Figure 4 and Figure 5:

As shown in Figure 3, when EEMD processes the simulation signal, it decomposes to obtain 10 layers of modal function, but by observing the spectrogram, only the first few layers are meaningful. We select the first five layers for observation. It can be seen that IMF1 and IMF5 extracted the signals with frequency of 280 Hz and 30 Hz successfully, but mode-mixing still occurs in IMF2 and IMF3. Compared with the MSSD decomposition method, the decomposition results of traditional EEMD has a lot of pseudo components, and the mode mixing is serious.

As shown in Figure 4, when SSD is used to process the simulated signal, the seven layer IMF is decomposed. Since the 7th and 8th layers are all meaningless noise signals, we select the first five layers for observation. It can be seen from the above figure that IMF1 and IMF5 extracted 280 Hz and 30 Hz signals successfully, but mode-mixing occurred in IMF2 and IMF3. The traditional SSD decomposition results still have serious mode mixing phenomenon, and although the ability of restrain pseudo component is improved compared to EEMD, it is still more.

As shown in Figure 5, the MSSD method decomposes and obtains three layers of IMF, and the frequency components of each component signal are successfully extracted. It is obvious that the mode mixing of the signal components extracted by MSSD has been greatly alleviated. The comparison results show that MSSD is better than the traditional SSD method and EEMD method in extracting fault features with noise.

## 4. Gearbox Composite Fault Simulation Signal Analysis

### 4.1. Construction of Simulation Signals

When the gearbox has composite faults, the vibration signals is often coexisting with multiple modulation sources. For this reason, in order to make the simulation analysis closer to the actual project, it is necessary to make the simulation signal more connected to the actual gearbox fault signal. In the construction of composite fault simulation signals, this article uses the simulation signals of common fault types of gearbox and rolling bearing faults for simulation analysis. The composition of the simulated signal x(t) is as follows:

(19) $$x(t)=x_{1}(t)+x_{2}(t)+x_{3}(t)+2.5\times randn(t)$$

**Figure 6:**
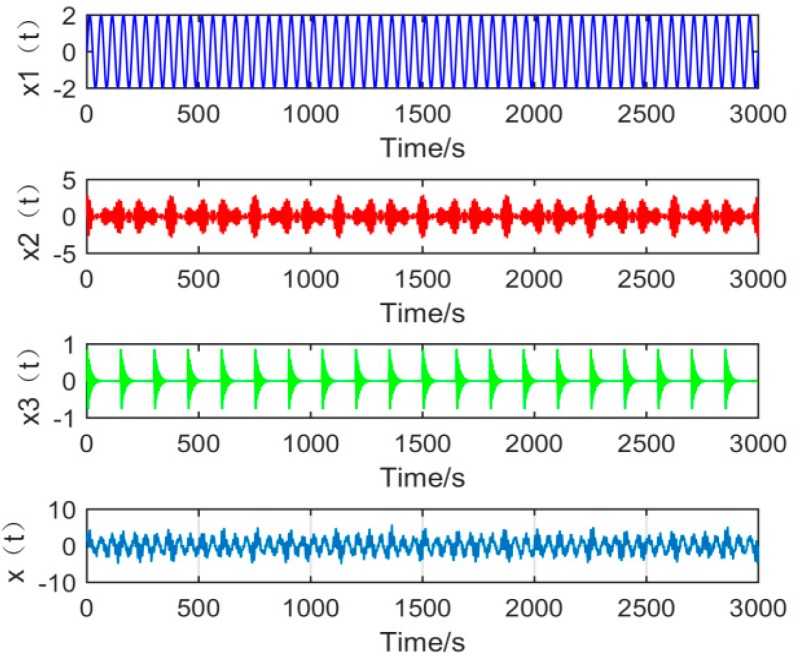
The time domain waveform of each component signal and simulation signal.

In the above formula, $x_{1}(t)=2\sin(2\pi f_{1}t)$ is a sine signal, and $x_{2}(t)=(1+\cos(2\pi f_{n1}t)+\cos(2\pi f_{n2}t))\sin(2\pi f_{z}t)$ is a gear fault simulation signal, $f_{n1}$ and $f_{n2}$ are the modulation frequencies of the modulation source, $f_{z}$ is the meshing frequency of the gears, and $x_{3}(t)=A_{m}\times \exp(-\frac{g}{T_{m}})\sin(2\pi f_{c}t)$ is a periodic impact signal, where $A_{m}$ represents the amplitude of the impact, g is the damping coefficient, $T_{m}$ is the cycle of shock, and $f_{c}$ is the rotation frequency of the bearing. The parameters are shown in the following Table 1:

Given the sampling points *N* to 3000 and the sampling frequency FS to 1500 Hz. The waveform of the signals $x_{1}(t)$, $x_{2}(t)$, and $x_{3}(t)$ and the time domain simulation signal *x*(*t*) are shown in Figure 6:

### 4.2. Comparison of Decomposition Results by Different Methods

**Figure 7:**
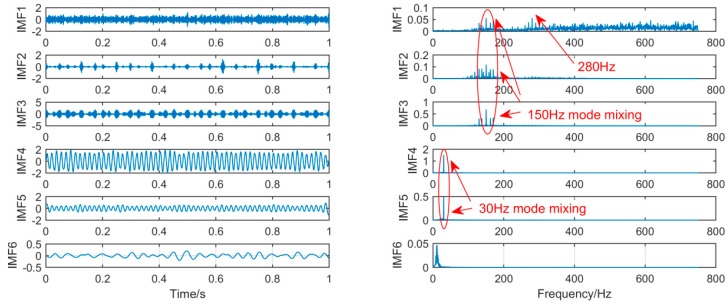
The spectrum of IMFs after CEEMDAN and its corresponding spectrum.

**Figure 8:**
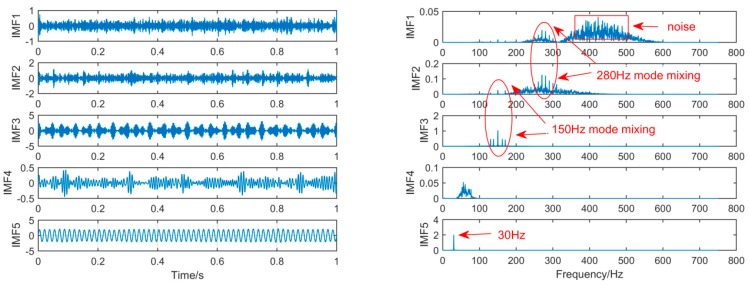
The spectrum of IMFs after SSD and its corresponding spectrum.

**Figure 9:**
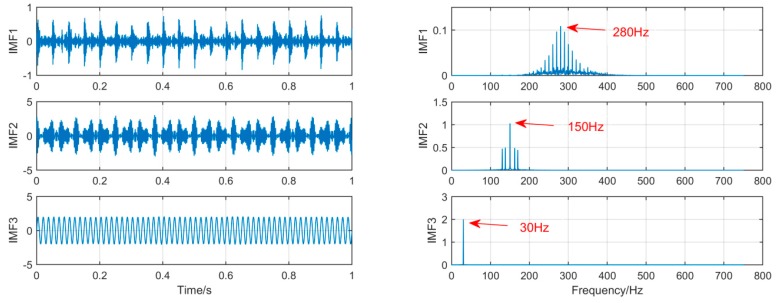
The spectrum of IMFs after MSSD and its corresponding spectrum.

In order to achieve the comparison of the decomposition results of different algorithms for the same simulation signal, this section will use the CEEMDAN, SSD, and MSSD methods to decompose the above-mentioned gearbox composite fault simulation signals. The decomposition results are shown in Figure 7, Figure 8 and Figure 9:

As shown in Figure 7, firstly, we choose the CEEMDAN decomposition method. For the result of CEEMDAN decomposition, we can observe from the above figure that there is a large amount of components. While the signals of frequency 280 Hz and 150 Hz are extracted in IMF1, IMF2, and IMF3 respectively, the spectral features of the signal are not obvious. The signal with a frequency of 30 Hz is decomposed into IMF4 and IMF5, it can be seen that mode mixing phenomenon is serious.

As shown in Figure 8, for the decomposition result of SSD, it can be found that a large amount of noise components occur in the IMF1 by observing the time spectrum, and mode-mixing also occurs in IMF1, IMF2, and IM3. Thus, in the components decomposed by the two decomposition methods CEEMDAN and SSD, a large amount of redundant noises are mixed, and the mode-mixing phenomenon is serious.

As shown in Figure 9, on the contrary, for the new method, by observing the time-frequency spectrum of the IMF obtained by MSSD, we can find that, in IMF3, the low-frequency component of 30 Hz in the original signal is successfully extracted, and its spectral features are very obvious, in IMF2, the 150 Hz center frequency of the amplitude modulation signal and the two modulation frequencies are also successfully extracted from the original signal, and in IMF1, it can be seen that the center frequency of 280 Hz and the 10 Hz sidebands distributed on both sides evenly are also very obvious. 

**Figure 10:**
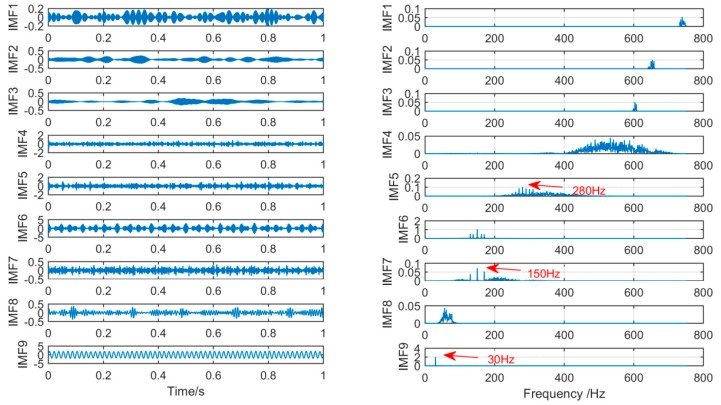
The spectrum of IMFs after SSD and its corresponding spectrum after an increase in the noise amplitude.

**Figure 11:**
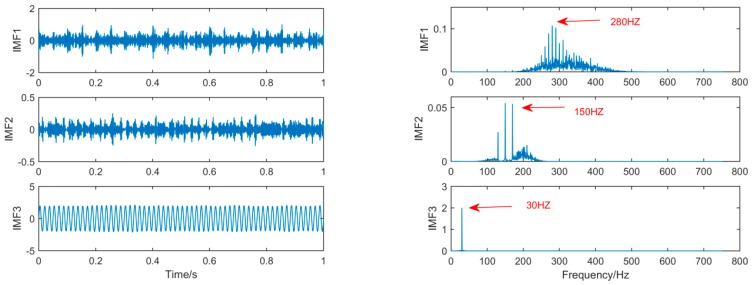
The spectrum of IMFs after MSSD and its corresponding spectrum after increase the noise amplitude.

Finally, we increase the noise amplitude by 0.5 on the original basis. Additionally, we compare the results of SSD and MSSD decomposition. The decomposition results are shown in Figure 10 and Figure 11:

As can be seen from the above figure, after increasing the amplitude of the noise, the MSSD still clearly decomposes the fault features that need to be extracted. Therefore, by comparing the decomposition results of several algorithms, it can be seen that in a noise environment, MSSD can not only effectively eliminate the mode-mixing phenomenon, but can also obtain the frequency features.

## 5. Gearbox Measured Signal Analysis

### 5.1. Gearbox Test Bench Design

In order to verify the effectiveness and feasibility of MSSD in engineering practice, this article conducted related tests on the closed power flow gearbox test bench. The composite fault vibration signals of the gearbox under normal, spalling failure of gear and bearing outer ring faults were measured. Then, we use the MSSD method to process these composite fault signals, and get a good effect. 

**Figure 12:**
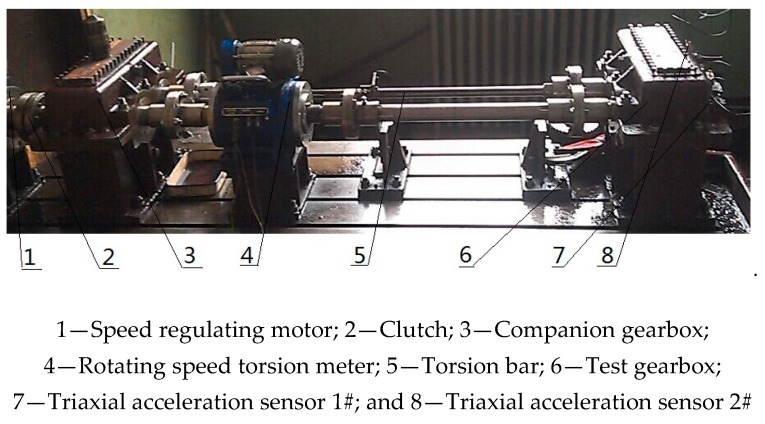
Gearbox test rig.

The experiment is a closed power flow test bench. The layout of the test bench is shown in Figure 12, in which the function of the torsion bar is to generate a load torque, thereby realizing the loading of the gearbox output shaft. The function of the speed regulating motor is to adjust the speed of the gearbox, and the speed range is 120–1200 r/min. 

Some experimental parameters of this experiment are shown in Table 2.

**Figure 13:**
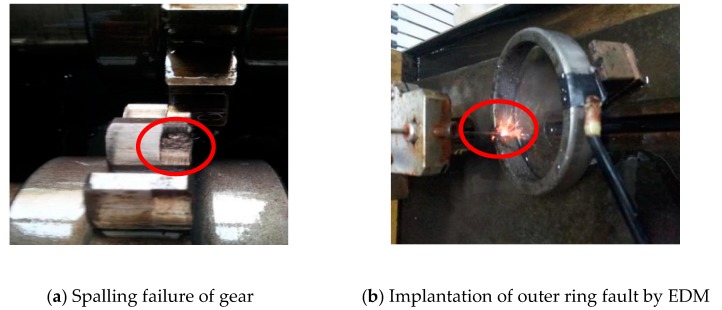
Figure of gear and bearing outer ring fault.

The type of fault includes spalling failure of gear, as shown in Figure 13a, and the outer ring fault obtained by EDM, as shown in Figure 13b.

### 5.2. Experimental Signal Analysis

When the gearbox generates a fault signal, in the experiment, the generated signal is first transmitted to the shaft, then transmitted to the bearing through the shaft, the sensor will fainlly receive the signal. The installation principle of acceleration sensor is that the position of the sensor and the signal source should be as close as possible. In this case, the signal features will be complete during the transfer, so the optimal position of the measuring point is the base of bearing. This experiment provides two acceleration sensors to measure the vibration signal. The signal in the experiment is derived from the acceleration sensor 1.

**Figure 14:**
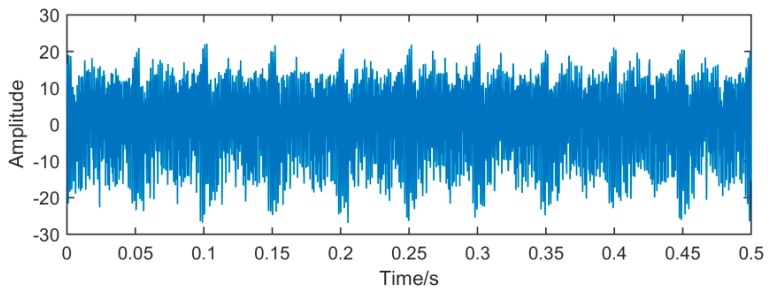
Time domain waveform of the fault signal.

**Figure 15:**
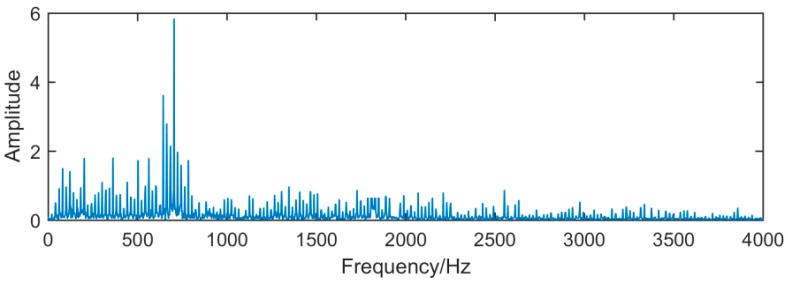
Frequency domain waveform of the fault signal.

**Figure 16:**
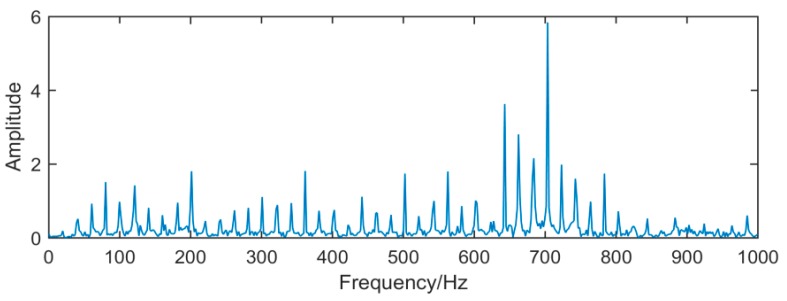
Amplified spectrum of Figure 15.

**Figure 17:**
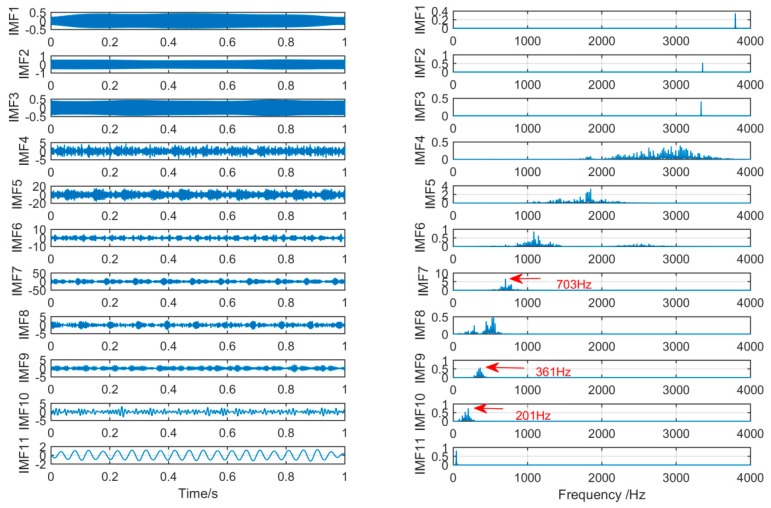
The spectrum of IMFs after SSD decomposition and its corresponding spectrum.

**Figure 18:**
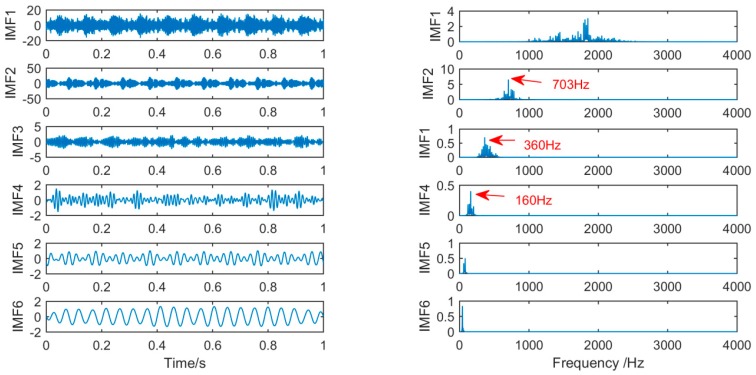
The spectrum of IMFs after MSSD decomposition and its corresponding spectrum.

**Figure 19:**
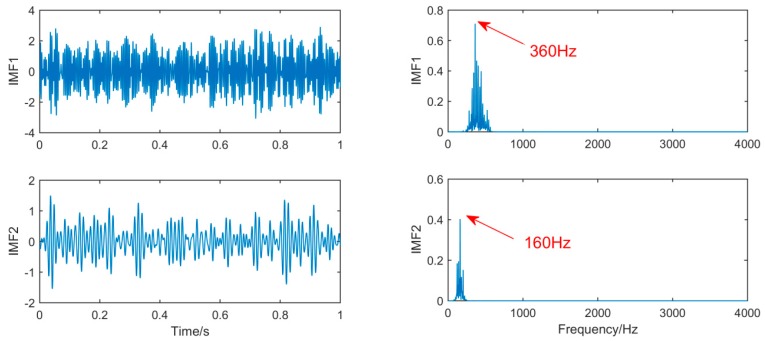
Fault features frequency of Figure 18.

It can be seen from Figure 14, Figure 15 and Figure 16, because of the noise, the waveform of the fault signal becomes disordered and irregular, Therefore, we use SSD and MSSD to decompose the above-mentioned gearbox composite fault signal, and extract the required fault frequency components for observation in the decomposition result, as shown in Figure 17, Figure 18 and Figure 19:

For the decomposition result of MSSD, by observing the spectrogram, it can be seen that in the gearbox fault signal, the fault frequency of the outer ring 160 Hz and the fault feature frequency of the gear 360 Hz are extracted, and the spectral features are very obvious. Subsequently, gears and balls were found to be slightly damaged when inspecting the gearbox, consistent with the analysis results. Overall, this method improves the SSD decomposition, and the decomposition results are significantly better than the SSD decomposition method in fault diagnosis.

## 6. Conclusions

In order to solve the problem that SSD is difficult to extract fault information of gearbox under strong noise and improve the efficiency of SSD decomposition, a new method, MSSD, is proposed to analyze composite signals. 

The advantage of MSSD is verified by decomposition of simulation signals. In addition, through experiments, it is found that MSSD method is suitable for the diagnosis of gearbox faults in experimental cases and practical engineering cases. The results show that MSSD can extract more fault information and reduce the interference of noise on fault diagnosis.

In future research, we will focus on the impact of various parameters on the final decomposition results and the optimization of the decomposition results of engineering measured signals.

## Figures and Tables

**Table 1:** The parameters of the simulation signal.

Parameter	$f_{1}$	$f_{n1}$	$f_{n2}$	$f_{z}$	$A_{m}$	g	$T_{m}$	$f_{c}$
**Numerical values**	30 Hz	12 Hz	20 Hz	150 Hz	2	0.1	0.1	280 Hz

**Table 2:** Experimental parameters.

Parameter	Numerical values
transmission ratio	1:1
engagement system	Half-tooth meshing
frequency of samplingFs	8000 Hz
Sampling point N	2000
load troque T	1000 N·m
Gear tooth number z	18
rotational speed n	1200 rpm
Rotor frequency $f_{n}$	20 Hz
Bearing outer ring fault frequency	160 Hz
Gear meshing frequency	360 Hz
